# Editorial: Short and long-term sequelae within the central nervous system due to COVID-19

**DOI:** 10.3389/fncel.2023.1146839

**Published:** 2023-02-07

**Authors:** Lucía Angélica Méndez-García, José Damián Carrillo-Ruiz, Helena Solleiro-Villavicencio

**Affiliations:** ^1^Laboratory of Immunometabolism, Research Division, General Hospital of Mexico Dr. Eduardo Liceaga, Mexico City, Mexico; ^2^Research Directorate, General Hospital of Mexico Dr. Eduardo Liceaga, Mexico City, Mexico; ^3^Department of Neurology and Neurosurgery, General Hospital of Mexico Dr. Eduardo Liceaga, Mexico City, Mexico; ^4^Facultad de Ciencias de la Salud, Universidad Anáhuac, Mexico City, Mexico; ^5^Posgrado en Ciencias Genómicas, Universidad Autónoma de la Ciudad de México, Mexico City, Mexico

**Keywords:** COVID-19, COVID-19 sequelae, neurologic effects of COVID-19, neurodegeneration, SARS-CoV-2, renin-angiotensin system, blood-brain barrier (BBB), long COVID syndrome

COVID-19 is caused by the severe acute respiratory syndrome coronavirus 2 (SARS-CoV-2) and has affected an estimated 664 million people with over 6.5 million deaths (Cumulative Confirmed COVID-19 Cases Deaths, 2023). Several neurologic symptoms are associated with the disease, from anosmia, headache, and dizziness, to stroke or seizure; these can remain long after the acute illness has passed (Baig, 2018; Zubair et al., 2020), causing a condition known as post-acute sequelae of SARS-CoV-2 infection (PASC) (Moghimi et al., 2021). It has been reported that PASC can lead to severe diseases such as encephalitis, encephalopathy, and meningitis. Unfortunately, little is known about the underlying molecular and cellular mechanisms around PASC; therefore, the therapeutic options to treat the neurological sequelae of COVID-19 are still scarce. For this reason, we hosted a Research Topic within Frontiers in Cellular Neuroscience, which aimed to analyze the cellular and molecular mechanisms that lead to short and long-term neurological COVID-19 sequelae, neuroinvasion mechanisms of SARS-CoV-2, and potential assessment and therapy for COVID-19-related neurological sequelae.

We received high-quality papers from researchers worldwide focused on different aspects of this Research Topic.

It is essential to explore the molecular mechanisms and signaling pathways deregulated after SARS-CoV-2 infection to understand the short- and long-term sequelae. In this sense, the renin-angiotensin signaling pathway (RAS) is pivotal during and after the infection. Accordingly, Méndez-García et al. contributed to this special issue to better understand how the binding of SARS-CoV-2 to its cellular receptor angiotensin-converting enzyme 2 (ACE2) causes the deregulation of RAS, leading to the accumulation of the pentapeptide angiotensin 2 (Ang-2) in cells and specific regions of the brain thus causing neurological damage. Moreover, in this review, the authors discuss the RAS-molecular targets that could be used for therapeutic purposes to treat the short and long-term neurological COVID-19-related sequelae.

Furthermore, it has been proposed that pre-existing neurological conditions such as Alzheimer's Disease (AD) could impact the development and severity of PASC. Hence, Zhao and Lukiw contributed to this Research Topic with an extraordinary opinion paper that points out hypothetical mechanisms that could explain why elderly-AD patients with COVID-19 have a worse prognosis and higher mortality. First, the viral spread into the central nervous system (CNS) may be due to the increased expression of ACE2 in neurons of the medulla oblongata and pons in the brainstem, containing the brain's medullary respiratory center; however, it is proposed that there might be an enrichment of this receptor in the limbic regions of AD patients, increasing their incidence and susceptibility to COVID-19. Also, they highlight that molecular modifications, such as the aggregation or misfolding of proteins (e.g., β-amyloid peptide) present in neurodegenerative diseases, could modulate the severity and the appearance of sequelae of the infection. Interestingly, authors also suggest that severely affected COVID-19 patients may be predisposed to developing neurodegenerative disorders, for instance, AD; this can occur due to detrimental viral effects on CNS and peripheral nervous system (PNS) at a structural, functional, and homeostatic level. Thus, recent studies suggest bidirectional effects between neurodegenerative diseases and COVID-19, which should be studied in greater depth.

Several studies have proposed that the neurological sequelae of COVID-19 are a consequence of systemic inflammation but not by direct infection of the virus to the brain, owing to the existence of a critical cerebral structure responsible for preventing the colonization of microorganisms and viruses, which is known as the blood-brain barrier (BBB). On this topic, Ju et al. reported the development of an *in vitro* BBB model composed of a brain endothelial cell line (HCMEC/D3), brain vascular pericytes (HBVP), and an astrocyte cell line (U87MG). Thus, for the first time, it was demonstrated that the SARS-CoV-2' envelope (S2E) protein inhibited the BBB cell viability in a dose- and time-dependent manner. In addition, the S2E viral protein caused damage to endothelial permeability by crossing the HCMEC/D3 cell monolayer, triggering the inflammatory response. This study demonstrates that not only is the cytokine storm responsible for the BBB disruption, but the virus *per se* can also cause this effect.

This Research Topic also focused on searching for tools to identify and evaluate the sequelae of long COVID. In this context, the most reported symptoms are fatigue, orthostatic symptoms, difficulty with attention and concentration (symptoms called “brain fog”), myalgias, and disrupted sleep, ranging from 2 to 40% of cases. To assess this symptomatology, Vernon et al. developed a smartphone app with which they applied the NASA Lean Test (NLT), an orthostatic stress test, to determine different symptoms, mainly hemodynamic and cognitive abnormalities. Results retrieved by the app were from people with long COVID or encephalomyelitis/chronic fatigue syndrome (ME/CFS) and compared to the ones derived from a control group. In the patients' groups (long-COVID and ME/CFS), the NLT showed a progressive narrowing in the pulse pressure, and the cognitive measures of reaction time worsened in these groups compared to the control group. Therefore, this smartphone app is a simple and helpful way to identify orthostatic intolerance and brain fog in PASC.

In conclusion, this Research Topic suggests that some factors that favor the CNS-viral invasion are the ubiquitous expression of ACE2 and the viral mechanism damaging the BBB's integrity, in which the S2E protein is involved. It also shows that the SARS-CoV-2 infection can deregulate systemic and brain RAS, leading to acute and chronic COVID-19-related neurological symptoms. On the other hand, this special issue explains that pre-existing neurodegenerative diseases can function as triggers for severe COVID-19 and immediate and long-term neurological symptoms. But also, severe COVID-19 could favor the development of some neurodegenerative diseases. Finally, a simple and easily accessible tool is shown to determine symptoms associated with long-term COVID-19. This highlights the importance of developing this type of application in terms of public health.

## Author contributions

HS-V and LM-G: writing—original draft. HS-V, LM-G, and JC-R: revising and supervising. All authors contributed to the article and approved the submitted version.
